# The Association between Serum Biomarkers and Disease Outcome in Influenza A(H1N1)pdm09 Virus Infection: Results of Two International Observational Cohort Studies

**DOI:** 10.1371/journal.pone.0057121

**Published:** 2013-02-27

**Authors:** Richard T. Davey, Ruth Lynfield, Dominic E. Dwyer, Marcello H. Losso, Alessandro Cozzi-Lepri, Deborah Wentworth, H. Clifford Lane, Robin Dewar, Adam Rupert, Julia A. Metcalf, Sarah L. Pett, Timothy M. Uyeki, Jose Maria Bruguera, Brian Angus, Nathan Cummins, Jens Lundgren, James D. Neaton, INSIGHT FLU 002 & 003 Study Groups

**Affiliations:** 1 National Institute of Allergy and Infectious Diseases, National Institutes of Health, Bethesda, Maryland, United States of America; 2 Infectious Disease Division, Minnesota Department of Health, St. Paul, Minnesota, United States of America; 3 Department of Virology, Centre for Infectious Diseases and Microbiology, Westmead Hospital and University of Sydney, Westmead, New South Wales, Australia; 4 HIV Unit, Department of Medicine, Hospital José María Ramos Mejía, Buenos Aires, Argentina; 5 Research Department of Infection and Population Health, University College London, London, England, United Kingdom; 6 Division of Biostatistics, University of Minnesota, Minneapolis, Minnesota, United States of America; 7 Virus Isolation and Serology Laboratory, SAIC-Frederick, Inc., Frederick National Laboratory, Frederick, Maryland, United States of America; 8 AIDS Monitoring Laboratory, SAIC-Frederick, Inc., Frederick, Maryland, United States of America; 9 Kirby Institute, Faculty of Medicine, University of New South Wales, Sydney, New South Wales, Australia; 10 Influenza Division, Centers for Disease Control and Prevention, Atlanta, Georgia, United States of America; 11 Servicio de Inmunocomprometidos, Hospital José María Ramos Mejía, Buenos Aires, Argentina; 12 Nuffield Department of Medicine, University of Oxford, Oxford, England, United Kingdom; 13 Division of Infectious Diseases, Mayo Clinic, Rochester, Minnesota, United States of America; 14 Department of Infectious Diseases, Copenhagen University Hospital/Rigshospitalet & University of Copenhagen, Copenhagen, Denmark; Karolinska Institutet, Sweden

## Abstract

**Background:**

Prospective studies establishing the temporal relationship between the degree of inflammation and human influenza disease progression are scarce. To assess predictors of disease progression among patients with influenza A(H1N1)pdm09 infection, 25 inflammatory biomarkers measured at enrollment were analyzed in two international observational cohort studies.

**Methods:**

Among patients with RT-PCR-confirmed influenza A(H1N1)pdm09 virus infection, odds ratios (ORs) estimated by logistic regression were used to summarize the associations of biomarkers measured at enrollment with worsened disease outcome or death after 14 days of follow-up for those seeking outpatient care (FLU 002) or after 60 days for those hospitalized with influenza complications (FLU 003). Biomarkers that were significantly associated with progression in both studies (p<0.05) or only in one (p<0.002 after Bonferroni correction) were identified.

**Results:**

In FLU 002 28/528 (5.3%) outpatients had influenza A(H1N1)pdm09 virus infection that progressed to a study endpoint of complications, hospitalization or death, whereas in FLU 003 28/170 (16.5%) inpatients enrolled from the general ward and 21/39 (53.8%) inpatients enrolled directly from the ICU experienced disease progression. Higher levels of 12 of the 25 markers were significantly associated with subsequent disease progression. Of these, 7 markers (IL-6, CD163, IL-10, LBP, IL-2, MCP-1, and IP-10), all with ORs for the 3^rd^ versus 1^st^ tertile of 2.5 or greater, were significant (p<0.05) in both outpatients and inpatients. In contrast, five markers (sICAM-1, IL-8, TNF-α, D-dimer, and sVCAM-1), all with ORs for the 3^rd^ versus 1^st^ tertile greater than 3.2, were significantly (p≤.002) associated with disease progression among hospitalized patients only.

**Conclusions:**

In patients presenting with varying severities of influenza A(H1N1)pdm09 virus infection, a baseline elevation in several biomarkers associated with inflammation, coagulation, or immune function strongly predicted a higher risk of disease progression. It is conceivable that interventions designed to abrogate these baseline elevations might affect disease outcome.

## Introduction

The sudden and unexpected emergence in 2009 and subsequent rapid global spread of a novel influenza virus, A(H1N1)pdm09, was yet another reminder of the ongoing challenges posed by this rapidly evolving class of respiratory viruses to world health [1], [2]. Both its seeming defiance early on of well-established patterns of seasonality, initial alarming reports of its heightened virulence in segments of the population (e.g. otherwise healthy non-elderly adults) not necessarily conforming to conventional risk groups, atypical clinical manifestations, and rapid emergence of A(H1N1)pdm09 virus as the major influenza virus causing human disease worldwide further augmented concerns about the potential threat of this novel virus to previous gains in prevention and management of this respiratory infection [3]. These concerns galvanized a global effort both to study and control A(H1N1)pdm09 virus infection through new and heightened surveillance, improved international communication, as well as the rapid development, testing, and deployment of vaccine strategies effective against the novel virus [4]-[6]. Fortunately, whether as a direct major consequence of these efforts alone or perhaps more as a combination of these efforts with still poorly understood elements of the biology of the virus itself, influenza A(H1N1)pdm09 virus has remained a major circulating human influenza virus but failed to reach the heights of morbidity and mortality previously feared. While largely still retaining its sensitivity to neuraminidase inhibitors, along the way it also assumed a more typical seasonal pattern of incidence and currently is considered a seasonal subtype of H1N1 [7]. Although recently supplanted as the predominant circulating virus in many parts of the Northern and Southern Hemispheres by influenza A (H3N2) as well as influenza B, A(H1N1)pdm09 remained detectable throughout 2011 influenza and 2012.

There has been a long-standing effort to identify and better characterize possible predictors of the severity of influenza virus infection in the human host [8]. In particular, it would be highly desirable for the treating clinician to have at hand a simple set of prognostic indicators, ideally both those that are readily obtainable from the patient and easily performed in the laboratory, that would help to guide therapy, as well as predict which subset of patients are potentially at greater risk for developing more serious sequelae from influenza infection following their initial diagnosis [9]. This is especially true if, as with A(H1N1)pdm09 virus infection, patient characteristics and the course of infection do not necessarily follow the typical clinical seasonal pattern for influenza. Furthermore, such prognostic markers may provide greater insight into which aspects of host defense are activated by the infection, and whether this activation is beneficial or harmful to the host. Ultimately, such insights may lead to better adjunctive therapy.

Both with A(H1N1)pdm09 virus infection and with disease due to other seasonal influenza subtypes, considerable interest has been generated in trying to characterize the potential role that cytokine dysregulation (so-called “cytokine storm”) triggered in the host may play in the pathogenesis and outcome of the viral infection [10]. This phenomenon includes the generation of markedly elevated levels of various cytokines and other pro-inflammatory mediators early in the course of natural infection that influence both the height and duration of the innate and adaptive immune response [11]. While overall the production of these mediators is felt to be protective, in some cases it is possible that an exaggerated or prolonged inflammatory response may actually contribute to a worsened disease outcome.

The INSIGHT H1N1v Outpatient study (FLU 002) and the INSIGHT H1N1v Hospitalization study (FLU 003) are two international observational cohort studies of influenza launched in 2009 whose purpose is to describe adult participants in geographically diverse locations who present for medical treatment due to influenza-like illness (ILI) and are documented to have laboratory-confirmed influenza and their outcomes over 14 days (FLU 002) and 60 days (FLU 003) of follow-up [12].

In this report we present the results of a comprehensive panel of serum biomarker determinations performed on blood specimens obtained at study entry in patients from these two cohorts with confirmed A(H1N1)pdm09 virus infection. Our goal was to study the association of the biomarkers with the risk of developing worsened disease outcomes as defined *a priori* in the two studies.

## Methods

### Ethics Statement

The FLU 002 and FLU 003 protocols were approved by the institutional review boards (IRB) or institutional ethics committees (IEC) at the University of Minnesota and at each of the other 63 participating clinical sites worldwide. Formal written documentation of IRB/IEC approval was required of each site Principal Investigator during the registration process that preceded site activation as a study center. Copies of these approval letters are filed with the central coordinating center at the University of Minnesota. All patients gave signed informed consent prior to enrollment.

### Study Design

The two international studies, FLU 002 and FLU 003, were initiated by the National Institutes of Health in August 2009 under the auspices of the INSIGHT (International Network for Strategic Initiatives in Global HIV Trials) clinical trials network. The INSIGHT network conducts these ongoing studies through a central coordinating center at the University of Minnesota and four international coordinating centers located in the United States (Washington DC), Europe (London and Copenhagen), and Australasia (Sydney). The study design and statistical considerations underlying each study were described previously [12]. Briefly, the two studies initially focused on the global manifestations of A(H1N1)pdm09 infection and together were designed to cover a broad clinical spectrum ranging from outpatients presenting with mild symptomatology (FLU 002) to those with more serious disease requiring hospitalization due to complications of influenza (FLU 003). The purpose of these studies is to estimate the percent of adult patients presenting with influenza-like illness (ILI) that is due to laboratory-confirmed influenza and to identify clinical and virologic factors associated with the risk of a worsened disease outcome or death. Each study takes advantage of an established international network of community and hospital-based investigators already in place and supplemented by additional sites seeing large numbers of adults diagnosed with acute influenza.

### Data Collection in FLU 002 and 003

At the time of enrollment the following information was collected: patient demographics, height, weight and vital signs; date of ILI onset, earliest contact with the health system for current illness; use of neuraminidase inhibitors to prevent or treat influenza in the preceding 14 days; medical history and underlying medical conditions, pregnancy status, smoking history, and current medications; influenza vaccination history since 2008 and pneumococcal vaccination history; and antiviral, antibacterial and other treatments prescribed at enrollment. Also recorded were local laboratory test results for influenza A; chest radiograph findings; and other local laboratory tests performed as part of standard of care.

In both studies respiratory (nasal and oropharyngeal) swabs were collected at enrollment and sent to one of two central laboratories to confirm by RT-PCR the local influenza diagnosis and to determine subtype. Identical methods were used by each laboratory. Serum samples were also collected from each participant at enrollment.

Patients enrolled in the outpatient study (FLU 002) with ILI were followed for 14 days for progression to hospitalization, the development of complications, or death. For the inpatient study (FLU 003) patients could be enrolled while in the general ward or in the intensive care unit (ICU). In either case patients were followed for 60 days. For those enrolled in the general ward, outcomes assessed included death, requirement for the ICU and/or mechanical ventilation, or prolonged hospitalization; the latter was defined as an inpatient stay exceeding 28 days of the 60-day follow-up period, not necessarily consecutively. For participants enrolled after already having been admitted to the ICU, death or prolonged hospitalization for >28 days were the primary outcomes.

### Biomarker Study

Patients were included in this study if they had influenza A(H1N1)pdm09 virus infection confirmed by RT-PCR at a central laboratory and had completed ascertainment of outcome status at day 14 and day 60 in the two studies, respectively. Serum samples obtained at enrollment were analyzed for all such patients in the inpatient study. For efficiency a nested case-control sampling scheme was used in the outpatient study, where samples were analyzed for each patient who experienced an event (28 patients) within the 14 day follow-up period and for three controls (patients who survived the 14 day follow-up period without complications or hospitalization and who were declared to be symptoms-free at day 14) matched on country of enrollment, age (± 5 years) and duration of symptoms. For two cases only 1 suitable control and for one case 2 controls were found (thus, a total of 79 controls instead of 84).

The blood samples were centrifuged and stored at -80 degrees Celsius until analysis. Sera concentrations of multiple cytokines and chemokines were obtained using a Pro-inflammatory 9-plex (IL-1 beta, IL-2, IL-6, IL-8, IL10, IL-12p70, GM-CSF, IFN-gamma, and TNF-alpha), a Chemokine 9-plex (Eotaxin, MIP-1beta, Eotaxin-3, TARC, IP-10, IL-8, MCP-1, MDC, and MCP-4), a Vascular Injury II Panel (CRP, VCAM-1, ICAM-1, and SAA), and LBP (Meso Scale Diagnostics, Gaithersburg, MD). Additional ELISA results were assayed for CRP, sCD14, sCD163, IL-6 (R&D Systems Inc., MN, USA). D-Dimer measurements were made using the VIDAS assay system (BioMerieux, Durham, NC). For biomarkers determined by both multiplex and ELISA, ELISA test results are cited. IL-8 was measured using both a chemokine and cytokine platform, only the latter was cited. Altogether, we report the results for 25 biomarkers. The sera samples for these studies were analyzed blinded to event status. Samples from outpatients and hospitalized patients were analyzed at the same time by the central laboratory at SAIC-Frederick.

While acknowledging that there is considerable overlap in cellular origin, cascading immunological relationships, and downstream effects among many of these biomarkers, for ease of comparison the 25 markers are displayed by broadly grouping them into four functional categories: 1) macrophage pro-inflammatory activation response; 2) acute phase response; 3) T cell activation response; and 4) macrophage chemokine response.

### Statistical analysis

The association of each of the 25 biomarkers with disease severity/progression was assessed in three separate analyses: 1) for disease severity, a cross-sectional comparison of biomarker levels for participants in FLU 002 versus FLU 003, and for those in FLU 003 enrolled in the general ward versus the ICU; 2) a comparison of biomarker levels for participants with disease progression after 14 days of follow-up versus those without progression in FLU 002 (case-control analysis); and 3) a comparison of biomarker levels for participants with disease progression after 60 days of follow-up versus those without progression in FLU 003 (cohort analysis). While power is greater for the cross-sectional comparisons than the follow-up comparisons within each study, a disadvantage of these comparisons is that the temporal association between the biomarker level and disease severity is not known (i.e., whether the biomarker is elevated as a consequence of disease severity or predicts disease progression is uncertain). This problem is overcome, at least in part, with the follow-up comparisons since in both FLU 002 and FLU 003 biomarker levels were determined prior to the disease progression outcome.

Simple summary statistics were use to describe the characteristics of patients in the two biomarker studies. To reduce the impact of outlying levels and to account for the positively skewed distribution of the biomarkers, results are categorized in tertiles and log_10_ transformed. The tertiles are defined separately for FLU 002 and FLU 003 [Table S1 in Supporting Information].

For the cross-sectional comparison, analysis of covariance with covariates corresponding to age and geographic location was used to compare the biomarker levels for patients in the two studies at the time of enrollment. Unlike the follow-up analyses within each study that are described below, we did not adjust for duration of symptoms as we considered it to be on the causal pathway for this cross-sectional comparison. For these comparisons, back-transformed levels (geometric means) are cited for each study. In FLU 003, further comparisons are made for the subgroups defined by location of enrollment (general ward versus ICU). For FLU 002, the log_10_ transformed biomarker levels for the controls and events were weighted to account for the larger FLU 002 group of patients from which the controls were selected for biomarker analyses.

For the follow-up comparisons in FLU 002 and FLU 003, logistic regression was used to summarize the association of each biomarker with the disease progression outcomes. For FLU 002 conditional logistic regression analyses for matched case–control studies were used. For FLU 003, unconditional logistic regression analyses stratified by location of enrollment (general ward versus ICU) and with covariates corresponding to the matching factors used in FLU 002 (age, duration of symptoms and geographic location) were carried out. Odds ratios (ORs) for the upper tertile versus the lowest tertile are cited along with 95% confidence intervals (CIs) and *p*-values. Analyses that used the log_10_ transformed biomarkers were also carried out; in the summary tables we refer to the p-values from this analysis as the p-value for linear trend. Analyses that further adjusted for race (black vs non-black), gender, current smoking, BMI and history of chronic conditions were also carried out and gave similar results. Kaplan-Meier survival curves are used to compare the patterns of mortality for tertiles of IL-6 in the FLU 003 study.

Two approaches were taken to minimize the risk of identifying false-positive associations between biomarkers and disease progression by highlighting subsets of individual biomarkers: 1) biomarkers that are significantly (p<0.05) related to disease progression based on the linear trend p-value in both studies; and 2) biomarkers which are significant (p≤ 0.002) in one study but not the other. The latter level of significance corresponds to a Bonferroni adjusted p-value (0.05/25).

To determine the relationship of multiple biomarkers with disease progression, we took advantage of the functional groupings that were identified. A global test procedure proposed by O’Brien for multiple endpoints is used [13]. With this approach, each marker in the raw scale within a functional grouping is ranked from lowest to highest, the ranks of the individual markers are summed for each patient. We refer to the sum of the ranks as the “biomarker score”. This biomarker score is then compared for patients who experienced an event versus those who did not with logistic regression models as described above (i.e., ORs for upper versus lowest tertile of the biomarker score are cited). Advantages of this procedure are simplicity and increased power if the biomarkers within a category all trend in the same direction. A disadvantage is that while the global test identifies biomarker groupings that are significant, it does not provide information on which markers are driving the statistical significance. Also, it is not an optimal test procedure if the association of some markers with disease outcome is positive and that of some other markers is negative. Thus, we also carried out a likelihood ratio test that tested the significance of adding all the markers (in the log_10_ scale) in a functional category to a base model that only included age, duration of symptoms and geographic region.

Statistical analyses were performed using SAS (Version 9.2).

## Results

**Figure 1:**
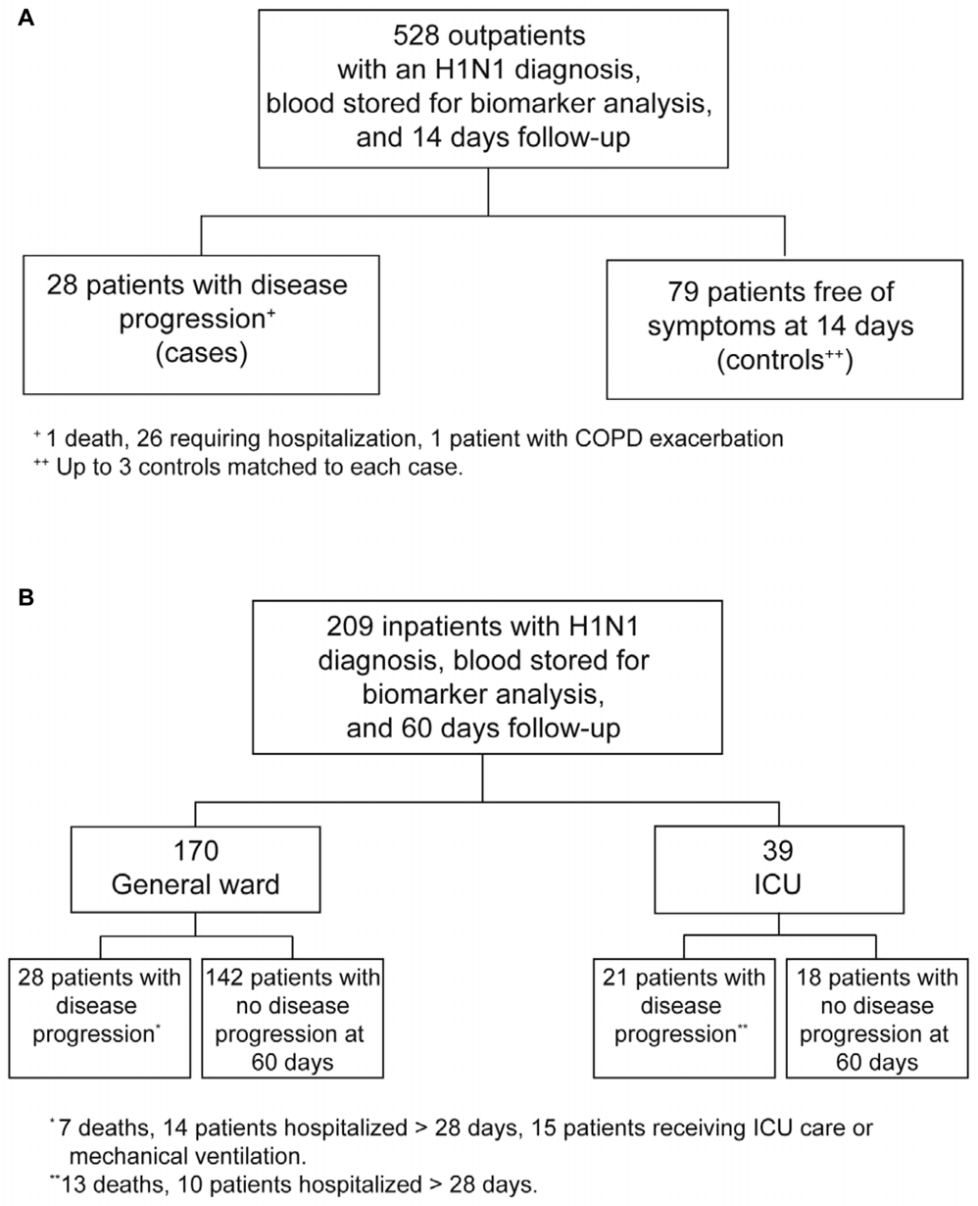
Biomarker study designs. The respective study designs and distribution of patients with and without severe disease outcomes in the outpatient case-control study FLU 002 (A) and in the hospitalization cohort study FLU 003 (B).

At the time of this biomarker investigation, 737 patients with influenza A(H1N1)pdm09 virus infection centrally confirmed by RT-PCR had completed ascertainment of outcome status in FLU 002 (528 patients) or FLU 003 (209 patients). Patients were enrolled from 63 sites in 14 countries. The risk of serious outcomes and the baseline characteristics varied considerably for participants in FLU 002 and FLU 003. Of the 528 patients with ILI seen at one of the outpatient clinics, 28 (5.3%) had disease progression requiring hospitalization (26 patients), one developed a severe complication (chronic obstructive pulmonary disease exacerbation), and one died within 14 days of enrollment (Figure 1A). These 528 patients were enrolled during two influenza seasons beginning in September 2009 in the Northern (n = 505) and Southern (n = 23) Hemispheres.

Of the 209 hospitalized patients enrolled in FLU 003, 170 had been enrolled while in the general ward and 39 from the ICU. These 209 patients were also enrolled during the same two influenza seasons in the Northern (n = 203) and Southern Hemispheres (n = 6). Overall, during the 60-day follow-up period, 49 (23.4%) of the 209 patients died, entered the ICU (if enrolled in the general ward), or had a hospital stay > 28 days. Figure 1B depicts these outcomes depending on whether patients were enrolled from the general hospital ward or from the ICU.

Table 1 summarizes the characteristics at study entry for participants enrolled in either FLU 002 or FLU 003 overall and, for FLU 003 participants, by location of enrollment (general ward or ICU). As expected, the median patient age of 30 years for the outpatient cohort was substantially lower than that of the hospitalized cohort, as was the duration of symptoms, the percentages of patients with an elevated BMI, who were pregnant, who had chronic medical co-morbidities, or who had received antiviral medication in the 14 day period prior to enrollment.

**Table 1:** Baseline Characteristics of the FLU 002 and FLU 003 Cohorts: Patients with Central Laboratory Confirmed A(H1N1)pdm09 Infection.

	FLU 002		FLU 003		
	Entire Cohort	Case-Control	Entire Cohort	General Ward	ICU
No. Patients	528	107	209	170	39
**Characteristics**					
Age – median (IQR)	30 (24, 40)	30 (25, 40)	49 (37, 61)	48 (37, 61)	51 (38, 59)
Female - %	51.5	56.1	47.8	52.9	25.6
Black race - %	6.1	8.4	4.3	4.1	5.1
BMI > 30 - %	6.4	12.9	23.0	23.8	19.4
Smoker - %	21.2	24.3	31.2	33.7	19.4
Pregnant* - %	1.8	5.0	15.7	17.0	0
Duration of symptoms – median days (IQR)	2 (1, 3)	2 (1, 3)	5 (3, 8)	5 (3, 7)	8 (5, 10)
2+ qualifying complications - %	N/A	N/A	34.0	29.4	53.8
Asthma or COPD - %	6.6	7.5	25.8	29.4	10.3
CVD, diabetes, liver or renal disease - %	1.9	0.9	26.8	24.7	35.9
HIV - %	7.8	11.2	3.3	2.9	5.1
Other immunosuppressive disease - %	0.8	0.9	9.6	9.4	10.3
Corticosteroids used to treat ILI - %	N/A	N/A	23.9	20.6	38.5
Taking statin - %	3.9	2.9	11.4	12.1	8.1
Took antiviral med w/in 14 days before enrollment - %	2.6	3.7	66.8	63.5	81.6

* Current or within past 2 weeks, among women aged ≤ 45 years.

### Cross-Sectional Relationship of Individual Biomarkers with Disease Severity

Geometric means for each of the 25 biomarkers were compared for outpatients in FLU 002 and inpatients in FLU 003 (overall and according to location of enrollment) [Table 2]. There was a strong correlation between levels of the individual biomarkers and the likely severity of illness of each cohort at time of enrollment: that is, most biomarkers were significantly lower in FLU 002 patients compared to FLU 003 patients and, among FLU 003 patients, biomarkers were generally lower in those enrolled while in the general ward as compared to the ICU. Three exceptions were IFN-γ, MDC and TARC for which the means were significantly higher for patients in FLU 002 compared to FLU 003 and for which levels were higher for patients in FLU 003 who were enrolled in the general hospital ward compared to the ICU. The mean level of IP-10 was also significantly higher in patients in FLU 002 compared to the overall mean level in FLU 003 patients, although higher in the ICU subcohort of the latter population.

**Table 2:** Geometric Means of Biomarkers for Patients in FLU 002 and FLU 003.

		FLU 003				
	FLU 002*	All	General Care	ICU	P-value**	P-value***
Macrophage Proinflammatory Activation Response						
IL-6 (pg/ml)	8.1	9.7	8.2	19.4	.09	<.001
sICAM-1 (ng/ml)	143	229	204	380	.003	.03
CD 163 (ng/ml)	468	825	774	1089	<.001	.004
IL-8 (pg/ml)	19.4	32.3	29.1	50.7	<.001	.005
IL-10 (pg/ml)	13.1	16.2	14.6	24.9	.08	.009
TNF-α (pg/ml)	11.2	13.4	12.4	18.7	.002	<.001
sCD14 (ng/ml)	2037	2395	2329	2706	.002	.05
IL-12 p70 (pg/ml)	2.83	3.44	3.49	3.24	.33	.79
IL-1β (pg/ml)	0.23	0.18	0.19	0.15	.57	.72
Acute Phase Response						
CRP (µg/ml)	16.2	51.7	46.1	85.0	<.001	.008
D-dimer (µg/ml)	0.88	1.30	1.08	2.84	<.001	<.001
SAA (µg/ml)	55	141	131	197	<.001	.13
LBP (µg/ml)	13.1	19.9	17.5	34.9	<.001	<.001
sVCAM-1 (ng/ml)	263	410	373	619	<.001	.002
T Cell Activation Response						
GM-CSF (pg/ml)	0.6	1.5	1.2	3.1	.02	.10
IL-2 (pg/ml)	1.4	2.3	2.1	3.7	.007	.003
IFN-γ (pg/ml)	6.4	1.9	2.0	1.5	<.001	.59
Macrophage Chemokine Response						
MCP-1 (pg/ml)	738	680	596	1201	.33	<.001
MCP-4 (pg/ml)	621	592	559	760	.51	.008
IP-10 (pg/ml)	1846	1267	1155	1900	.001	.01
MIP-1β (pg/ml)	144	119	117	131	.009	.36
Eotaxin (pg/ml)	986	970	963	1005	.81	.71
Eotaxin-3 (pg/ml)	14.1	17.6	16.7	21.7	.11	.11
MDC (pg/ml)	3704	2405	2470	2140	<.001	.16
TARC (pg/ml)	371	238	253	182	<.001	.05

* The levels in FLU 002 were weighted to take into account sampling controls from a cohort of 528 patients.

** ANOVA for differences in log_10_ biomarker, FLU 002 vs FLU 003.

*** ANOVA for difference in log_10_ biomarker, general ward vs ICU enrollment in FLU 003.

### Relationship of Individual Biomarkers with Disease Progression during Follow-up

Table 3 compares characteristics for patients in each study who had disease progression with those who did not. In FLU 002 there was a trend for more cases than controls to be of black race (p = 0.06). However, none of the differences between cases and controls reached statistical significance. In FLU 003, patients with subsequent disease progression were more likely to have a longer duration of clinical symptoms prior to enrollment (p = 0.02) and to use antiviral medications in the 14 day period prior to enrollment (p = 0.03). As expected, the most striking difference between those who progressed and those who did not was the difference in risk for those enrolled in the general ward versus those enrolled from the ICU (p<0.001).

**Table 3:** Baseline Characteristics of Patients in the FLU 002 and FLU 003 Biomarker Cohorts According to Event Status.

	FLU 002			FLU 003*		
	Cases	Controls	P-value**	Events	No Events	P-value***
No. Patients	28	103		49	160	
**Characteristics**						
Age – median (IQR)	31 (26, 45)	30 (25, 39)	0.14	50 (40, 61)	49 (35, 60)	.31
Female - %	64.3	52.4	0.33	36.7	51.3	.43
Black race - %	17.9	4.9	0.06	4.1	4.4	.85
BMI > 30 - %	19.2	10.1	0.15	15.8	24.7	.30
Smoker - %	21.4	30.1	0.73	26.7	32.5	.81
Pregnant**** - %	11.1	1.9	0.53	0	17.8	.96
Duration of symptoms – median days (IQR)	2 (1, 3.5)	2 (1, 3)	N/A	8 (5, 10)	5 (3, 7)	.02
2+ qualifying complications - %	N/A	N/A	N/A	46.9	30.0	.20
Asthma or COPD - %	14.3	3.9	0.19	16.3	28.8	.32
CVD, diabetes, liver or renal disease - %	3.6	0.0	0.99	36.7	23.8	.17
HIV - %	17.9	8.9	0.33	6.1	2.5	.31
Other immunosuppressive disease - %	3.6	0.0	0.99	16.3	7.5	.06
Corticosteroids used to treat ILI - %	N/A	N/A	N/A	30.6	21.9	.64
Taking statin - %	7.1	1.0	0.18	2.2	14.0	.06
Took antiviral med w/in 14 days before enrollment - %	3.6	2.9	0.93	83.3	61.9	.03
Enrolled from the ICU - %	N/A	N/A	N/A	42.9	11.3	<.001

* Entire FLU003 biomarker cohort independent of site of enrollment (general ward versus ICU).

** From fitting a conditional logistic regression model.

*** From fitting a logistic regression model stratified by type of unit at enrollment (general ward versus ICU).

**** Current or within past 2 weeks, among women aged ≤ 45 years.

Tables 4 and 5 give median values at entry for those who progressed and those who did not in FLU 002 and FLU 003, respectively. Also cited are ORs for the upper versus lowest tertile of each marker, the p-value corresponding to this comparison, and the p-value for linear trend (from a model that includes the log-transformed level of the biomarker). Results were similar after further controlling for race (black vs non-black), gender, current smoking, BMI, and history of chronic conditions (data not shown).

**Table 4:** FLU 002 Patients: Odds Ratios of Progression for the Highest Versus Lowest Tertile of Each Biomarker.

	Cases		Controls		Odds Ratio*			
Biomarker	Median	IQR	Median	IQR	OR	95% CI	P-value	P-value**
Macrophage Proinflammatory Activation Response								
IL-6 (pg/ml)	17.1	9.8, 26.1	8.1	5.1, 14.7	4.44	1.35, 14.6	.01	.002
sICAM-1 (ng/ml)	143	93, 274	159	85, 282	1.01	0.30, 3.38	.98	.70
CD 163 (ng/ml)	586	494, 893	497	387, 650	2.74	0.88, 8.53	.08	.01
IL-8 (pg/ml)	20.0	11.4, 44.6	13.9	10.0, 22.9	2.68	0.87, 8.30	.09	.08
IL-10 (pg/ml)	15.3	11.2, 29.7	10.9	7.6, 16.8	3.92	1.18, 13.0	.03	.02
TNF-α (pg/ml)	11.0	9.7, 13.8	10.3	8.3, 13.2	2.94	0.87, 9.95	.08	.29
sCD14 (ng/ml)	2520	1878, 3207	2010	1660, 2516	3.05	0.99, 9.35	.05	.08
IL-12 p70 (pg/ml)	3.49	1.57, 4.41	1.86	1.22, 4.72	2.74	0.86, 8.74	.09	.44
IL-1β (pg/ml)	0.74	0.43, 1.43	0.58	0.23, 1.03	2.83	0.84, 9.57	.09	.77
Acute Phase Response								
CRP (µg/ml)	25.2	9.5, 73.7	18.9	6.2, 34.8	2.10	0.68, 6.44	.20	.12
D-dimer (µg/ml)	1.22	0.70, 1.72	0.71	0.42, 1.31	4.82	1.44, 16.2	.01	.06
SAA (µg/ml)	76	27, 202	62	25, 136	1.33	0.42, 4.23	.63	.40
LBP (µg/ml)	18.8	12.8, 32.0	12.9	8.8, 20.8	3.80	1.17, 12.4	.03	.03
sVCAM-1 (ng/ml)	258	175, 477	303	165, 525	1.03	0.32, 3.31	.96	.90
T Cell Activation Response								
GM-CSF (pg/ml)	1.1	0.7, 3.1	0.9	0.3, 2.5	2.26	0.65, 7.90	.20	.30
IL-2 (pg/ml)	2.8	1.4, 4.8	1.6	1.1, 2.8	7.71	1.63, 36.5	.01	.03
IFN-γ (pg/ml)	9.0	3.6, 18.9	7.7	4.3, 11.6	1.48	0.47, 4.63	.50	.14
Macrophage Chemokine Response								
MCP-1 (pg/ml)	897	613, 1335	696	501, 1181	2.48	0.80, 7.62	.11	.03
MCP-4 (pg/ml)	701	429, 912	695	436, 980	0.66	0.20, 2.21	.51	.90
IP-10 (pg/ml)	3454	1981, 5496	1853	1058, 2807	4.53	1.44, 14.2	.010	.003
MIP-1β (pg/ml)	142	107, 202	132	101, 237	1.17	0.40, 3.43	.77	.36
Eotaxin (pg/ml)	922	652, 1538	1001	786, 1400	0.89	0.32, 2.47	.83	.98
Eotaxin-3 (pg/ml)	18.5	13.2, 26.8	15.3	11.4, 21.0	2.99	0.91, 9.84	.07	.06
MDC (pg/ml)	3754	2687, 4980	3389	2758, 4979	0.94	0.29, 3.03	.92	.52
TARC (pg/ml)	324	208, 460	342	221, 561	0.53	0.15, 1.80	.31	.19
No. patients	28		79					

* Odds ratio & p-value for highest vs lowest tertile, from conditional logistic model with matching on age, duration of symptoms and country of enrollment.

** As above, p-value for log_10_-transformed biomarker.

**Table 5:** FLU 003 Patients: Odds Ratios of Progression for the Highest Versus Lowest Tertile of Each Biomarker.

	Events		Non-events		Odds Ratio*			
Biomarker	Median	IQR	Median	IQR	OR	95% CI	P-value	P-value**
Macrophage Proinflammatory Activation Response								
IL-6 (pg/ml)	22.1	13.5, 27.2	9.5	3.9, 17.8	6.14	1.86, 20.3	.003	.004
sICAM-1 (ng/ml)	513	277, 729	241	101, 375	6.00	2.02, 17.8	.001	<.001
CD 163 (ng/ml)	1251	824, 1916	650	512, 961	5.55	2.08, 14.8	<.001	<.001
IL-8 (pg/ml)	52.0	24.1, 85.3	22.4	13.9, 46.6	3.97	1.44, 10.9	.008	.001
IL-10 (pg/ml)	25.0	13.0, 69.9	10.5	6.7, 21.1	7.23	2.61, 20.0	<.001	<.001
TNF-α (pg/ml)	15.5	13.0, 24.6	12.1	9.2, 15.7	3.92	1.29, 11.9	.02	.002
sCD14 (ng/ml)	2990	2397, 3698	2249	1761, 2995	3.36	1.30, 8.68	.01	.03
IL-12 p70 (pg/ml)	2.56	1.14, 9.09	2.77	1.58, 6.69	1.09	0.45, 2.59	.85	.65
IL-1β (pg/ml)	0.52	0.25, 1.36	0.42	0.17, 0.95	1.83	0.73, 4.55	.19	.98
Acute Phase Response								
CRP (µg/ml)	124	52.2, 203	46.4	22.7, 101	3.98	1.47, 10.8	.007	.008
D-dimer (µg/ml)	3.07	1.54, 4.78	1.09	0.55, 1.86	3.21	1.21, 8.48	.02	<.001
SAA (µg/ml)	285	95, 494	187	65, 390	1.59	0.66, 3.88	.30	.13
LBP (µg/ml)	39.9	22.6, 73.9	16.9	10.5, 30.3	4.46	1.68, 11.9	.003	.006
sVCAM-1 (ng/ml)	800	536, 1152	394	181, 650	6.69	2.26, 19.8	<.001	<.001
T Cell Activation Response								
GM-CSF (pg/ml)	3.2	2.1, 6.3	2.4	0.9, 4.2	2.09	0.81, 5.42	.13	.06
IL-2 (pg/ml)	3.8	2.0, 7.6	1.9	1.2, 3.6	2.81	1.12, 7.08	.03	.005
IFN-γ (pg/ml)	3.2	1.4, 8.7	2.6	1.1, 6.3	3.81	1.39, 10.4	.009	.35
Macrophage Chemokine Response								
MCP-1 (pg/ml)	1105	579, 2498	546	363, 804	4.04	1.55, 10.5	.004	<.001
MCP-4 (pg/ml)	550	385, 754	577	389, 886	0.48	0.18, 1.25	.13	.31
IP-10 (pg/ml)	2486	954, 7175	990	532, 1935	3.73	1.48, 9.44	.005	<.001
MIP-1β (pg/ml)	107	81, 165	118	82, 163	0.94	0.38, 2.33	.90	.90
Eotaxin (pg/ml)	980	567, 1667	981	651, 1384	0.92	0.39, 2.16	.85	.95
Eotaxin-3 (pg/ml)	17.0	13.6, 29.7	16.1	11.1, 27.2	1.34	0.50, 3.55	.56	.13
MDC (pg/ml)	1821	1346, 2524	2525	1879, 3430	0.26	0.10, 0.71	.009	.02
TARC (pg/ml)	149	93, 245	251	139, 439	0.25	0.09, 0.72	.010	.01
No. patients	49		160					

* Odds ratio & p-value for highest vs lowest tertile, adjusted for enrollment unit (ICU vs general ward), age, symptom duration, and continent of enrollment.

** P-value for log_10_ biomarker, adjustment as above.

**Figure 2:**
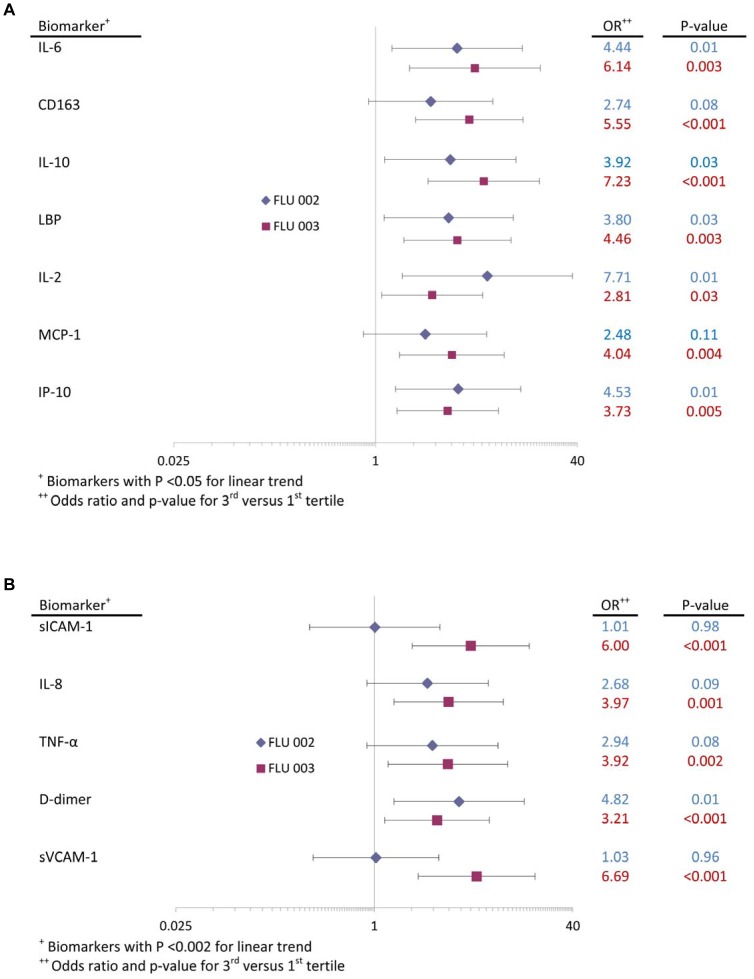
Biomarkers significantly related to disease progression. The biomarkers found to be significantly associated with disease progression in both studies (A) and in FLU 003 only (B) are shown. Odds ratios (3^rd^/1^st^ tertile) and 95% confidence intervals are depicted.

Higher levels of 12 markers were significantly associated with disease progression. Seven markers were significant (p< 0.05) in both studies: IL-6, CD163, IL-10, LBP, IL-2, MCP-1, and IP-10. For ease of comparison, Figure 2A gives the ORs (3^rd^/1^st^tertile) and p-values for these 7 biomarkers for each study. All of the ORs in Figure 2A are > 2.4. Five other biomarkers were significant (p = 0.002 or smaller) in FLU 003 and not significant (p> 0.05) in FLU 002 (Figure 2B). Of note, for FLU 002 none of the 12 biomarkers were significant at a Bonferroni-adjusted level of significance of 0.002 or lower with the exception of IL-6 (OR = 4.4).

**Figure 3:**
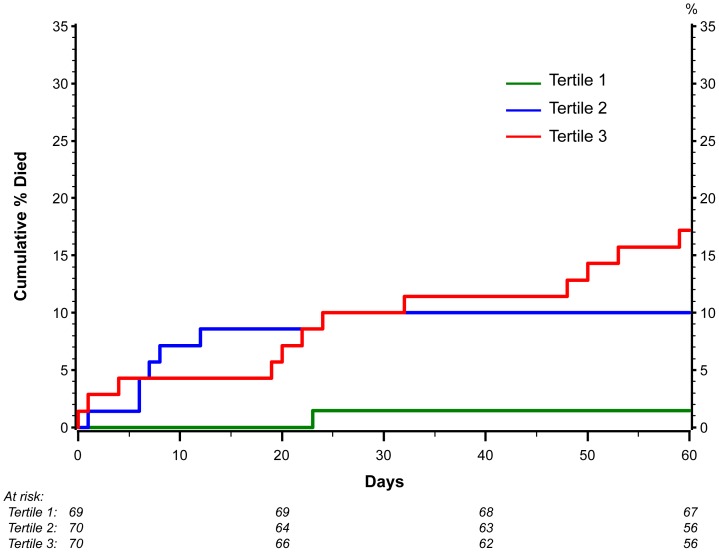
Mortality by tertiles of IL-6 in the FLU 003 study. A Kaplan-Meier graph of cumulative mortality (%) for FLU 003 participants according to baseline levels of IL-6 as grouped into tertiles.

For inpatients (FLU 003), we also examined the relationship of IL-6 with mortality (Figure 3). Only one death occurred among patients in the lowest tertile (<6.4 pg/mL) at enrollment; in contrast, for the 2^nd^ and 3^rd^tertiles (6.4–17.3 and >17.3 pg/mL), 7 and 12 deaths occurred (p = 0.008 for difference in mortality among the tertiles).

### Relationship of Functional Categories of Biomarkers with Disease Progression

**Figure 4:**
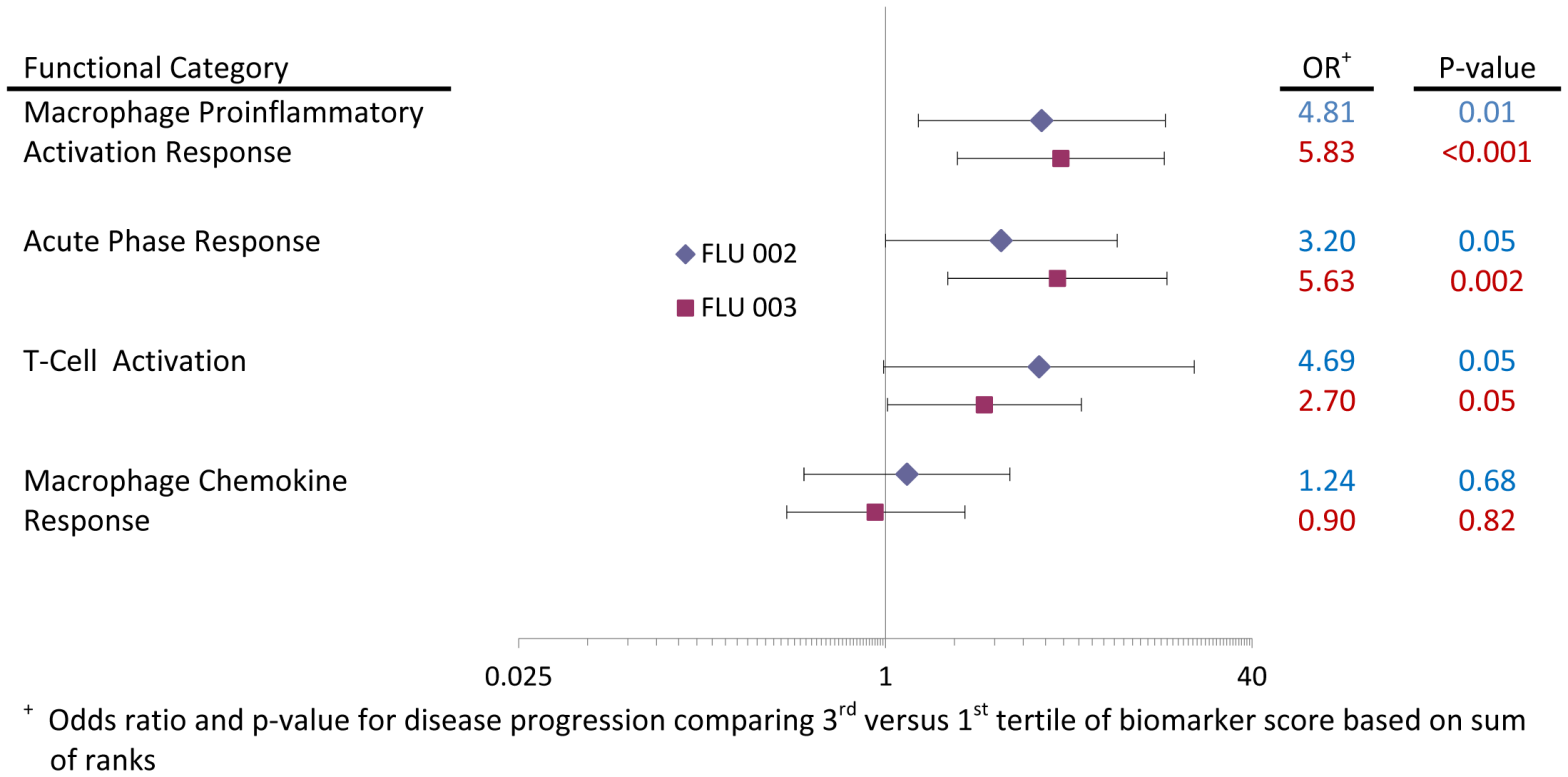
Relationship of functional categories of biomarkers to disease progression in FLU 002 and FLU 003. The odds ratios and 95% confidence intervals for the risk of disease progression in FLU 002 (in blue) and FLU 003 (in red) are depicted according to four functional categorizations of the 25 biomarkers analyzed in these two studies.

Figure 4 summarizes ORs (3^rd^/1^st^tertile) for the biomarker score for each functional grouping within each study (see Table 2 for biomarkers included in these four groupings). For both studies the first three functional categories showed stronger relationships (ORs were 2.7 or greater) with disease progression than did the last (macrophage chemokine response). In part, the weaker association of this last category with disease progression was due to some biomarkers in that grouping having a positive association with disease progression and others displaying an inverse association. To investigate this further, we carried out a logistic regression analysis for each study in which we compared the chi-square statistic resulting from adding the last category of biomarkers to a model that only included age, duration of symptoms and geographic region. For FLU 003, the last category of markers (macrophage chemokine response) contributed significantly (p<0.001) to model fit (data not shown) as did the first three categories, macrophage proinflammatory activation response (p<0.001), acute phase response (p<0.001), and T cell activation response (p = 0.03). For FLU 002, macrophage proinflammatory activation response (p = .003) and macrophage chemokine response (p = .02) were significant determinants of disease progression. The other two categories were of borderline significance (p-values  = 0.06 and 0.07).

## Discussion

*In vitro* laboratory experiments, small animal model testing, and clinical studies of humans with acute influenza, including those infected with A(H1N1)pdm09 virus, have revealed a number of cytokines and other mediators that appear to play a significant role in the initial host response to influenza virus infection. In mouse or ferret studies experimental infections with virulent strains of influenza A viruses have been associated with higher elevations in select biomarkers such as IL-6, TNF-α, and IFN-α and have usually been associated with worsened disease outcomes in those animals [14], [15]. In an influenza challenge study reported in 1998 in which normal human volunteers were experimentally infected with a seasonal influenza A (H1N1 Texas/36/91) virus, Hayden et al. found that both IL-6 and IFN-alpha levels in nasal lavage fluids peaked early in the course of infection and correlated directly with viral titers, temperature, mucus production, and symptom scores [8]. Several other clinical studies in adults or children have been reported by investigators in which the addition of various serum or nasal biomarker level measurements to the standard clinical evaluation appeared to add to the diagnostic certainty of respiratory virus infection with seasonal influenza [16]–[19].

In a number of generally cross-sectional studies focusing either partially or exclusively upon confirmed cases of A(H1N1)pdm09 virus infection in specific geographic areas, potential correlations between various cytokine levels and disease severity have been reported. Investigators from Mexico reported that A(H1N1)pdm09 virus infection resulted in stronger *in vitro* upregulation of IL-6, CCL3, and CXCL8 in 72-hour cell cultures as well as elevated serum levels of IL-6, CXCL8, and certain other cytokines in individuals infected with this subtype compared to those with seasonal influenza virus infection [20]. In separate publications a group in Hong Kong reported that elevated levels of IL-6, CXCL8, CCL2, and sTNFR-1 correlated with severe cases of A(H1N1)pdm09 virus infection overall and, in particular, with the extent and severity of influenza-associated pneumonia [21], [22]. Similar findings concerning elevated IP-10 and IL-6 levels in cases of pediatric pneumonia were reported from Korea [23]. At least two groups from mainland China have described similar relationships for several of the pro-inflammatory cytokines withA(H1N1)pdm09 virus infection, and separate groups from Spain, Italy, and Romania have also described correlations between elevated levels of various cytokines such as IL-6, IL-15, and TNF-α and the severity of disease outcomes in patients with confirmed A(H1N1)pdm09 virus infection [24]–[28]. Most recently, a group from Canada has found IL-6 to be an important feature of the host response in both humans and mice infected with A(H1N1)pdm09 virus and, in the former case, found elevated IL-6 levels to be an important predictor of severe disease [29].

Biomarker analyses from our two large ongoing international studies of influenza described here have strengthened and extended these observations in important ways. A major virtue of the present studies is that these data were collected prospectively according to a common data-set and with defined periods of follow-up to assess disease progression, samples have been garnered from a relatively large number of patients living in geographically disparate regions of the world and analyzed through common central laboratory systems using standardized methodologies, and both studies included enrollments of patients with different severities of A(H1N1)pdm09 virus infection spanning over more than a single influenza season in those areas. Further, the samples were collected, shipped, and processed for analysis using identical procedures to minimize any potential artifactual effects on serum levels of the biomarkers measured.

Even after adjustments for multiple potentially confounding variables within each dataset according to different stringencies, there are several biomarkers whose baseline levels appear to be highly correlated with a worsened disease course according to the definitions of disease progression predefined for each study. Several could have been singled out in this context. However, the seven markers that were significantly correlated with disease progression in both studies were IL-6, CD163, IL-10, LBP, IL-2, MCP-1, and IP-10, a somewhat disparate set spanning all four functional groupings. While cross-sectional comparisons between studies must always be interpreted with caution, for six of these seven markers, the exception being MCP-1, the absolute levels of each at study entry also appeared to correlate with disease severity at time of enrollment, as reflected principally by their higher geometric mean values in hospitalized patients versus outpatients.

Two notable inconsistences between the cross-sectional and follow-up findings were the associations with IFN-γand IP-10. Both markers were lower in the cross-sectional comparison of FLU 003 participants compared to FLU 002 participants, but within the two studies higher, not lower, levels were associated with an increased risk of disease progression during follow-up. The reason for this discrepancy remains unclear at present; however, the difference may reflect a limitation of cross-sectional comparisons in which temporal relationships are uncertain.

As a biomarker with known involvement in the pro-inflammatory cascade associated with many different types of infections, as well as one that has featured prominently in earlier other published analyses of the potential role of biomarkers in predicting influenza disease outcomes, we also chose to validate the strong predictive potential of IL-6 in these two studies. For both outpatients and those requiring hospitalization, serum IL-6 was a strong predictor of disease progression. For the hospitalized patients in FLU 003, those with an IL-6 level in the upper two tertiles were also at an increased of mortality. This is similar to prior observations a decade earlier in a small number of fatal cases of H5N1 infection [30]. A causal explanation for this is not fully elucidated, although animal data do support an association of elevated levels of IL-6 production with enhanced lethality of the infecting virus [31], [32].

Although the strong statistical associations found in these two studies between select individual biomarkers and a worsened disease outcome are compelling, nonetheless these results present an obvious difficulty with extrapolation to the clinical arena at the present time. Most of the biomarkers described here are part of a multiplex testing array generally performed in a research setting and are not a routine part of the diagnostic work-up performed for a typical patient presenting with signs and symptoms of acute influenza. Hence, at present they may be of more value in providing insight into potential mechanisms of viral pathogenesis and host defense rather than in offering direct clinical benefit. There are some potential exceptions to this. D-dimer and CRP assays, for example, are generally available today in most acute care facilities as indicators of recent thrombotic events and abnormal systemic inflammation, respectively, and the test results are generally available in real time.

It is fair to say that, at present, there does not appear to be a single discrete biomarker readily available to the physician at the time of presentation that one can conclude adds unequivocably to the ability of the standard diagnostic assessment to predict the likelihood of disease progression in all patients. Nonetheless, as these multiplex assays become cheaper and more readily available beyond the research setting, this situation may improve. For example, assuming an exaggerated or dysfunctional cytokine response to acute influenza may actually contribute to disease severity in some cases, their additional value may be in pinpointing areas of this response that may be amenable to dampening or other forms of therapeutic intervention. Thus, at the same time that one is treating the virus in at-risk individuals, it is conceivable that adjunctive therapy directed at abrogating or redirecting an overly exuberant host response could also be introduced to further improve prognosis.

Although this leap from the research setting to the bedside still remains a challenge for the reasons cited, it is still reasonable to ask whether in the future even stronger prognostic utility or power might be found in analyzing select combinations or subsets of these markers, whether identified through statistical modeling or *de novo* as biologically plausible groupings. In addition to allowing for more intelligent utilization of available biomarkers, such an approach might further minimize justifiable concerns over potential over-interpretation of statistical associations seen in comparatively large batteries of single assay results not adjusted for multiple comparisons, such as those generated by the multiplex platforms in increasingly common use. In a limited look at this possibility, we found that combinations of multiple biomarkers from even four relatively indiscrete functional groupings did generate odds ratios for disease outcomes that were statistically robust, generalizable across both studies, and comparable to the most potent of the single biomarkers studied. The fact that elevated levels of certain biomarkers were found in each of the four groupings suggests a very broad and diverse immunologic response to acute influenza. But with further dissection of specific categories of markers it may be possible to delineate more precisely what role the disparate portions of the host response play in modulating the course of infection. Whether with further exposition such a combination approach might prove to be either more powerful or, ideally, disease-specific than any of the single biomarkers highlighted remains yet to be determined. In any case, these preliminary results are intriguing and provide a rationale for performing more detailed analyses of these particular biomarkers as additional patients are accrued in these studies and additional progression events recorded. It is certainly possible, for example, that combinations of other biomarkers, or even different logical groupings of the same biomarkers into other categories based upon such factors as common cellular origin or relative positioning within the pro-inflammatory cascade, might offer results of equal or superior prognostic value or provide even greater insight into potentially deleterious aspects of the host immune response.

The focus of this present analysis has been on the prognostic value of baseline biomarkers in predicting the subsequent course of disease specifically in A(H1N1)pdm09 virus-infected patients. Since both studies are ongoing on a multi-year basis in both the Northern and Southern Hemispheres, have been broadened to include patients presenting with all major subtypes of seasonal virus in circulation at the time, and now collect serial blood specimens for up to 60 days following enrollment, the intention is that these analyses can be expanded in at least two ways. One goal will be to map the kinetics in serum and plasma of each of the major biomarkers, singly and in groupings, from baseline through the course of acute infection and until the time of resolution and recovery. Surprisingly little is known about how these markers change differentially over time according to such factors as disease severity or the host’s baseline immune status, or how quickly they return to the pre-infection state. A second goal will be to compare the prognostic value of biomarkers in infection due to A(H1N1)pdm09 virus to that found in infection with other influenza viruses. It is unclear at present whether these correlations observed with A(H1N1)pdm09 virus infection reflect broad-based host response pathways that can be applied, for example, universally to influenza A virus infections as a whole, or whether more discrete differences in various cytokine response patterns will emerge as different subtypes are examined further. These and other types of analyses should be readily possible under the bounds of these ongoing studies.

## Supporting Information

Table S1 * The upper and lower tertiles for each of the biomarkers are defined separately for the FLU 002 and FLU 003 cohorts; these biomarkers are shown grouped into the four functional categories as defined in the main manuscript.
(DOCX)

## References

[1] Centers for Disease Control and Prevention Outbreak of swine-origin influenza A (H1N1) virus infection – Mexico, March–April 2009. (2009) MMWR Morb Mortal Wkly Rep 58: : 467–470. 19444150

[2] Novel Swine-Origin Influenza A (H1N1) Virus Investigation Team (2009) Emergence of a novel swine-origin influenza A (H1N1) virus in humans. N Engl J Med 360: 2605–2615. 19423869 10.1056/NEJMoa0903810

[3] Writing Committee of the WHO Consultation on Clinical Aspects of Pandemic (H1N1) 2009 Influenza (2010) Clinical aspects of pandemic 2009 influenza A (H1N1) virus infection. N Engl J Med. 362: 1708–19. 20445182 10.1056/NEJMra1000449

[4] Briand S, Mounts A, Chamberland M (2011) Challenges of global surveillance during an influenza pandemic. Public Health 125: 247–56. 21524774 10.1016/j.puhe.2010.12.007 PMC7111716

[5] Jain S, Kamimoto L, Bramley AM, Schmitz AM, Benoit SR, et al (2009) Hospitalized patients with 2009 H1N1 influenza in the United States, April-June 2009. N Engl J Med 361: 1935–44. 19815859 10.1056/NEJMoa0906695

[6] Greenberg ME, Lai MH, Hartel GF, Wichems CH, Gittleson C, et al (2009) Response to a monovalent 2009 influenza A (H1N1) vaccine. N Engl J Med 361: 2405–13. 19745216 10.1056/NEJMoa0907413

[7] World Health Organization website. Available: http://www.who.int/mediacentre/news/statements/2010/h1n1_vpc_20100810/en/index.html. Accessed 10 Aug 2010.

[8] Hayden FG, Fritz R, Lobo MC, Alvord W, Strober W, et al (1998) Local and systemic cytokine responses during experimental human influenza A virus infection. Relation to symptom formation and host defense. J Clin Invest 101: 643–9. 9449698 10.1172/JCI1355 PMC508608

[9] Ison MG, de Jong MD, Gilligan KJ, Higgs ES, Pavia AT, et al (2010) End points for testing influenza antiviral treatments for patients at high risk of severe and life-threatening disease. J Infect Dis 201: 1654–62. 20423224 10.1086/652498 PMC12821767

[10] Tisoncik JR, Korth MJ, Simmons CP, Farrar J, Martin TR, et al (2012) Into the eye of the cytokine storm. Microbiol Mol Biol Rev 76: 16–32. 22390970 10.1128/MMBR.05015-11 PMC3294426

[11] Fukuyama S, Kawaoka Y (2011) The pathogenesis of influenza virus infections: the contributions of virus and host factors. Curr Opin Immunol 23: 481–6. 21840185 10.1016/j.coi.2011.07.016 PMC3163725

[12] Dwyer DE (2011) The INSIGHT Influenza Study Group (2011) Surveillance of illness associated with pandemic (H1N1) 2009 virus infection among adults using a global clinical site network approach: the INSIGHT FLU 002 and FLU 003 studies. Vaccine 29 Suppl 2B56–62. 21757105 10.1016/j.vaccine.2011.04.105 PMC3703507

[13] O’Brien PC (1984) Procedures for Comparing Samples with Multiple Endpoints. Biometrics 40: 1097–87. 6534410

[14] Siye Wang, TrongQuang Le, Naoki Kurihara, Junji Chida, Youssouf Cisse, et al (2010) Influenza Virus—Cytokine-Protease Cycle in the Pathogenesis of Vascular Hyperpermeability in Severe Influenza. J Infect Dis 202: 991–1001. 20731583 10.1086/656044 PMC7537608

[15] Kang YM, Song BM, Lee JS, Kim HS, Seo SH (2011) Pandemic H1N1 influenza virus causes a stronger inflammatory response than seasonal H1N1 influenza virus in ferrets. Arch Virol 156: 759–67. 21234768 10.1007/s00705-010-0914-7

[16] Sumino KC, Walter MJ, Mikols CL, Thompson SA, Gaudreault-Keener M, et al (2010) Detection of respiratory viruses and the associated chemokine responses in serious acute respiratory illness. Thorax 65: 639–44. 20627924 10.1136/thx.2009.132480 PMC3018337

[17] Hutchinson AF, Black J, Thompson MA, Bozinovski S, Brand CA, et al (2010) Identifying viral infections in vaccinated Chronic Obstructive Pulmonary Disease (COPD) patients using clinical features and inflammatory markers. Influenza Other Respi Viruses 4: 33–9. 10.1111/j.1750-2659.2009.00113.x PMC4941951 20021505

[18] Sato M, Hosoya M, Wright PF (2009) Differences in serum cytokine levels between influenza virus A and B infections in children. Cytokine 47: 65–8. 19497758 10.1016/j.cyto.2009.05.003

[19] Heltzer ML, Coffin SE, Maurer K, Bagashev A, Zhang Z, et al (2009) Immune dysregulation in severe influenza. J Leukoc Biol 85: 1036–43. 19276177 10.1189/jlb.1108710 PMC2698588

[20] Zúñiga J, Torres M, Romo J, Torres D, Jiménez L, et al (2011) Inflammatory profiles in severe pneumonia associated with the pandemic influenza A/H1N1 virus isolated in Mexico City. Autoimmunity 44: 562–70. 21838592 10.3109/08916934.2011.592885

[21] Lee N, Wong CK, Chan PK, Chan MC, Wong RY, et al (2011) Cytokine response patterns in severe pandemic 2009 H1N1 and seasonal influenza among hospitalized adults. PLoS One 6: e26050. 22022504 10.1371/journal.pone.0026050 PMC3192778

[22] Lee N, Chan PK, Wong CK, Wong KT, Choi KW, et al (2011) Viral clearance and inflammatory response patterns in adults hospitalized for pandemic 2009 influenza A(H1N1) virus pneumonia. Antivir Ther 16: 237–47. 21447873 10.3851/IMP1722

[23] Kim YH, Kim JE, Hyun MC (2011) Cytokine response in pediatric patients with pandemic influenza H1N1 2009 virus infection and pneumonia: comparison with pediatric pneumonia without H1N1 2009 infection. Pediatr Pulmonol 46: 1233–9. 21626718 10.1002/ppul.21496 PMC7167952

[24] Shen HH, Hou J, Chen WW, Bai BK, Wang HB, et al (2011) Immunologic changes during Pandemic (H1N1) 2009, China. Emerg Infect Dis 17: 1053–5. 21749768 10.3201/eid1706.100643 PMC3358185

[25] Liu Y, Chen H, Sun Y, Chen F (2012) Antiviral role of Toll-like receptors and cytokines against the new 2009 H1N1 virus infection. Mol Biol Rep 39: 1163–72. 21603856 10.1007/s11033-011-0846-7

[26] Estella A (2011) Cytokine levels in bronchoalveolar lavage and serum in 3 patients with 2009 Influenza A(H1N1)v severe pneumonia. J Infect Dev Ctries 5: 540–3. 21795823 10.3855/jidc.1618

[27] Agrati C, Gioia C, Lalle E, Cimini E, Castilletti C, et al (2010) Association of profoundly impaired immune competence in H1N1v-infected patients with a severe or fatal clinical course. J Infect Dis 202: 681–9. 20670171 10.1086/655469

[28] Hagau N, Slavcovici A, Gonganau DN, Oltean S, Dirzu DS, et al (2010) Clinical aspects and cytokine response in severe H1N1 influenza A virus infection. Crit Care 14: R203. 21062445 10.1186/cc9324 PMC3220006

[29] Paquette SG, Banner D, Zhao Z, Fang Y, Huang SS, et al (2012) Interleukin-6 Is a Potential Biomarker for Severe Pandemic H1N1 Influenza A Infection. PLoS One 7: e38214. 22679491 10.1371/journal.pone.0038214 PMC3367995

[30] de Jong MD, Simmons CP, Thanh TT, Hien VM, Smith GJ, et al (2006) Fatal outcome of human influenza A (H5N1) is associated with high viral load and hypercytokinemia. Nat Med 12: 1203–7. 16964257 10.1038/nm1477 PMC4333202

[31] Murphy EA, Davis JM, McClellan JL, Carmichael MD, Rooijen NV, et al (2011) Susceptibility to infection and inflammatory response following influenza virus (H1N1, A/PR/8/34) challenge: role of macrophages. J Interferon Cytokine Res 31: 501–8. 21352081 10.1089/jir.2010.0143

[32] Wang S, Le TQ, Kurihara N, Chida J, Cisse Y, et al (2010) Influenza virus-cytokine-protease cycle in the pathogenesis of vascular hyperpermeability in severe influenza. J Infect Dis 202: 991–1001. 20731583 10.1086/656044 PMC7537608

