# Supplementary material for: The Association between Serum Biomarkers and Disease Outcome in Influenza A(H1N1)pdm09 Virus Infection: Results of Two International Observational Cohort Studies
Source: PLoS One. 2013 Feb 27;8(2):e57121. doi: 10.1371/journal.pone.0057121 (PMC3584122; doi:10.1371/journal.pone.0057121)
Supplement: Table S1 — * The upper and lower tertiles for each of the biomarkers are defined separately for the FLU 002 and FLU 003 cohorts; these biomarkers are shown grouped into the four functional categories as defined in the main manuscript. (DOCX) [file pone.0057121.s001.docx]

**Table S1:** Tertiles for Biomarkers in FLU 002 and FLU 003 *

|  | | | | |
| --- | --- | --- | --- | --- |
|  | **FLU 002** | | **FLU 003** | |
|  | **Lower** | **Upper** | **Lower** | **Upper** |
| **Macrophage Proinflammatory Activation Response** | | | | |
| IL-6 (pg/ml) | 6.5 | 14.6 | 6.4 | 17.3 |
| sICAM-1 (ng/ml) | 102 | 230 | 175 | 369 |
| CD 163 (ng/ml) | 442 | 608 | 593 | 994 |
| IL-8 (pg/ml) | 11.3 | 20.1 | 17.2 | 42.2 |
| IL-10 (pg/ml) | 9.2 | 15.5 | 9.0 | 19.9 |
| TNF-α (pg/ml) | 9.5 | 12.5 | 10.7 | 15.3 |
| sCD14 (ng/ml) | 1826 | 2450 | 2025 | 2863 |
| IL-12 p70 (pg/ml) | 1.50 | 3.95 | 1.83 | 4.77 |
| IL-1β (pg/ml) | 0.44 | 0.83 | 0.26 | 0.79 |
| **Acute Phase Response** | | | | |
| CRP (μg/ml) | 10.4 | 30.0 | 35.2 | 92.7 |
| D-dimer (μg/ml) | 0.61 | 1.11 | 0.79 | 1.89 |
| SAA (μg/ml) | 35 | 105 | 95 | 320 |
| LBP (μg/ml) | 10.8 | 18.2 | 13.4 | 30.5 |
| sVCAM-1 (ng/ml) | 206 | 440 | 277 | 662 |
| **T Cell Activation Response** | | | | |
| GM-CSF (pg/ml | 0.7 | 2.0 | 1.7 | 3.7 |
| IL-2 (pg/ml) | 1.3 | 2.4 | 1.6 | 3.4 |
| IFN-γ (pg/ml) | 5.2 | 11.0 | 1.4 | 5.2 |
| **Macrophage Chemokine Response** | | | | |
| MCP-1 (pg/ml) | 576 | 966 | 445 | 804 |
| MCP-4 (pg/ml) | 491 | 831 | 445 | 725 |
| IP-10 (pg/ml) | 1590 | 2683 | 677 | 1809 |
| MIP-1β (pg/ml) | 116 | 175 | 92 | 143 |
| Eotaxin (pg/ml) | 824 | 1172 | 729 | 1223 |
| Eotaxin-3 (pg/ml) | 13.3 | 19.5 | 12.9 | 22.7 |
| MDC (pg/ml) | 3033 | 4457 | 1912 | 2933 |
| TARC (pg/ml) | 260 | 423 | 149 | 316 |
